# Voltage Dependence of Proton Pumping by Bacteriorhodopsin Mutants with Altered Lifetime of the M Intermediate

**DOI:** 10.1371/journal.pone.0073338

**Published:** 2013-09-03

**Authors:** Sven Geibel, Èva Lörinczi, Ernst Bamberg, Thomas Friedrich

**Affiliations:** 1 Max-Planck-Institute of Biophysics, Department of Biophysical Chemistry, Frankfurt am Main, Germany; 2 Technical University of Berlin, Institute of Chemistry, Berlin, Germany; University of Leeds, United Kingdom

## Abstract

The light-driven proton pump bacteriorhodopsin (BR) from *Halobacterium salinarum* is tightly regulated by the [H^+^] gradient and transmembrane potential. BR exhibits optoelectric properties, since spectral changes during the photocycle are kinetically controlled by voltage, which predestines BR for optical storage or processing devices. BR mutants with prolonged lifetime of the blue-shifted M intermediate would be advantageous, but the optoelectric properties of such mutants are still elusive. Using expression in *Xenopus* oocytes and two-electrode voltage-clamping, we analyzed photocurrents of BR mutants with kinetically destabilized (F171C, F219L) or stabilized (D96N, D96G) M intermediate in response to green light (to probe H^+^ pumping) and blue laser flashes (to probe accumulation/decay of M). These mutants have divergent M lifetimes. As for BR-WT, this strictly correlates with the voltage dependence of H^+^ pumping. BR-F171C and BR-F219L showed photocurrents similar to BR-WT. Yet, BR-F171C showed a weaker voltage dependence of proton pumping. For both mutants, blue laser flashes applied during and after green-light illumination showed reduced M accumulation and shorter M lifetime. In contrast, BR-D96G and BR-D96N exhibited small photocurrents, with nonlinear current-voltage curves, which increased strongly in the presence of azide. Blue laser flashes showed heavy M accumulation and prolonged M lifetime, which accounts for the strongly reduced H^+^ pumping rate. Hyperpolarizing potentials augmented these effects. The combination of M-stabilizing and -destabilizing mutations in BR-D96G/F171C/F219L (BR-tri) shows that disruption of the primary proton donor Asp-96 is fatal for BR as a proton pump. Mechanistically, M destabilizing mutations cannot compensate for the disruption of Asp-96. Accordingly, BR-tri and BR-D96G photocurrents were similar. However, BR-tri showed negative blue laser flash-induced currents even without actinic green light, indicating that Schiff base deprotonation in BR-tri exists in the dark, in line with previous spectroscopic investigations. Thus, M-stabilizing mutations, including the triple mutation, drastically interfere with electrochemical H^+^ gradient generation.

## Introduction

Bacteriorhodopsin (BR) from *Halobacterium salinarum* acts as a light-driven proton pump. It generates an electrochemical gradient for protons, which then is utilized for secondary active transport processes and ATP synthesis. The BR transport mechanism is closely coupled to the photocycle, in which, after absorption of a photon, the retinal isomerizes from *all-tran*s to 13*-cis*, and via the spectroscopically distinct intermediates J, K and L the blue-shifted M intermediate is formed. The key steps in the BR photocycle for the directed, ‘vectorial’ transport process are the de- and reprotonation of the Schiff base (i.e. the formation of the M_1_ intermediate, the transition from M_1_ to the intermediate M_2_, and decay of M_2_). During the formation of M_1_, the Schiff base (SB) releases a proton to Asp-85, and its pK_a_ shifts from >13 to <4. Still in M_1_, the SB changes its pK_a_ back to >13, and accessibility changes from extracellular to intracellular during the M_1_→M_2_ transition. Subsequently, the retinal SB is reprotonated by Asp-96 from the intracellular side, whereby M_2_ relaxes back to the ground state *BR* via the photocycle intermediates N and O. The M→*BR* decay contains the rate-limiting and main electrogenic steps at depolarizing voltages [1].

Substantial for understanding the pump mechanism of BR was the determination of the protein’s structure in the ground state *BR* and several photocycle intermediates [2], [3], [4], [5], [6], [7], [8], [9], [10], [11], [12], [13]. Two experimental approaches were used, first, shock-freezing of crystals formed from BR wild-type protein immediately after illumination with actinic light [9], [11], second, crystallization of BR mutants, which accumulate certain photocycle intermediates. Mutants BR-D96N and BR-D96G, due to their drastically slowed M decay in spectroscopic experiments, were used for structural analysis of the M intermediate [7], [8], [10], [11], and mutants BR-F171C and BR-F219L were chosen for the structure of the N intermediate, [4], [5], [12].

**Figure 1:**
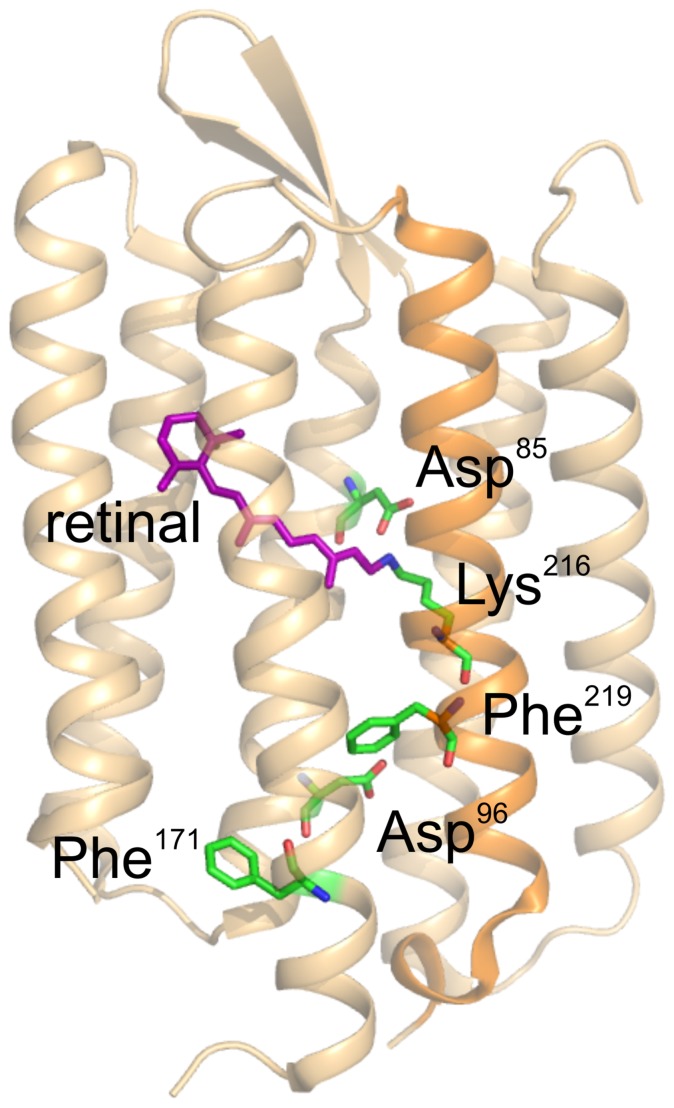
3D structure of BR. Cartoon representation of the 3D structure of bacteriorhodopsin according to the coordinates in PDB structure entry 1C3W by Luecke et al. (1999) prepared with PyMol 1.0 software. The retinal chromophore (magenta) is covalently linked via a Schiff base to Lys-216 in helix G (orange), which - together with the primary proton acceptor (Asp-85) and proton donor group (Asp-96) - is depicted in ball-and-chain representation (oxygen atoms: red, carbon atoms: green, nitrogen atoms: blue). Also shown are Phe-171 and Phe-219, which, together with Asp-96, were mutated herein.

The three-dimensional structure of BR-WT reveals seven α-helices (termed A-G) forming a transmembrane pathway for protons, which is subdivided into a cytoplasmic (CP) and an extracellular (EC) part by the chromophore retinal. The retinal is bound via a SB to Lys-216 in helix G (Figure 1). BR mutant structures showed that during the first half of the photocycle (*BR*→M) only minor structural changes occur, while the relaxation from the state M_1_ (with SB accessibility still towards EC) through M_2_ (with SB accessibility towards CP) back to *BR* is accompanied by distinct, albeit small, structural rearrangements. In detail, after excitation of the retinal, the CP part of the channel still assumes a closed conformation, but deprotonation of the SB and deprotonation of Asp-96 trigger movements of helices B, G and F, which open the CP part of the proton pathway [5], [7], [8], [9], [11], [12]. These studies revealed that well-concerted conformational rearrangements take place during the BR photocycle.

The mutant BR-D96G/F171C/F219L (BR-tri) which combines several of the above mutations was shown to exhibit structural features of the M intermediate already in the dark [10], [11]. In contrast to BR-WT and other structurally investigated BR mutants, BR-tri showed no detectable structural changes during the reaction cycle, although its proton pump activity was reported to reach 66% of BR-WT [14]. These findings have challenged the functional importance of structural changes for the efficiency of proton pumping by BR.

Besides extensive research that is currently devoted to the application of other retinylidene proteins such as channel- or halorhodopsins in optogenetics, there is a long-standing interest from biotechnology in the archetypical BR and its sequence variants, since BR is endowed with outstanding robustness against harsh natural or experimental conditions and can be considered to be the most thoroughly studied membrane transporter protein in biophysical research. The photochromic and optoelectric properties made it particularly interesting for biomolecular optoelectronic high-density or high-speed data processing and storage concepts, such as optical memories [15], holographic storage devices [16], [17], document security applications [18] or ultrafast optical switch logics [19], [20], [21]. For most of these applications, BR mutants with extended lifetimes of photointermediates with large photochromic shifts are desired that enable safe optical switching between quasi-stable spectral species. Promising variants would be BR mutants such as L93A stabilizing the red-shifted O intermediate [22], the aforementioned Asp-96 substitutions stabilizing the blue-shifted M intermediate, or F171C and F219L destabilizing M in favor of N, which are all well characterized by crystallography. However, despite the wealth of spectroscopic and structural data, very little information about the crucial function of these mutants is available, which is light-driven H^+^ transport across cellular membranes. Of particular interest is the study of the transmembrane potential’s influence on the photocycle kinetics, which can only be achieved by heterologous expression in cell lines that facilitate electrophysiology. To close this gap, we expressed mutants BR-D96N, BR-D96G, BR-F171C, BR-F219L, and BR-D96G/F171C/F219L in *Xenopus* oocytes and studied the voltage dependence of transient and stationary photocurrents using the two-electrode voltage-clamp technique (TEVC). These experiments reveal the influence of mutations and negative membrane potentials on proton pumping. In combination with an analysis of the lifetime of M intermediates in the respective mutants allowed us to derive general conclusions about the regulation of proton pumping by the lifetime of M, as exemplified previously for wild-type BR [1], [23], [24].

## Results

**Figure 2:**
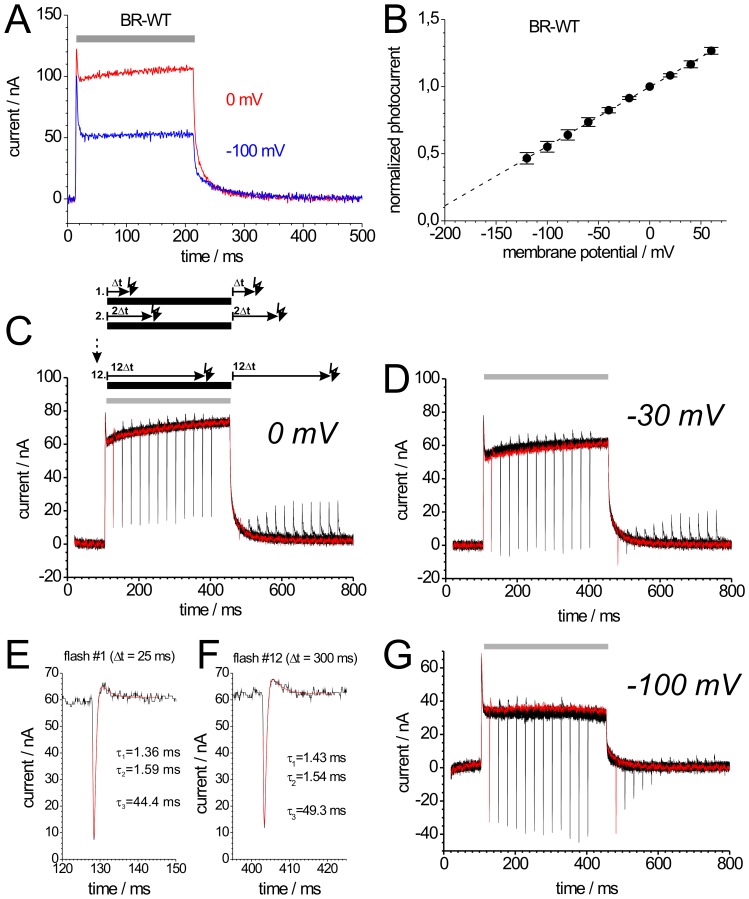
Properties of BR-WT stationary and transient photocurrents. Photocurrents of BR-WT expressed in *Xenopus* oocytes are shown, which were evoked either by continuous illumination with green light (A) and/or blue laser flashes (C,D,G). (A) BR-WT photocurrents induced by illumination with green light (grey bar) at 0 mV (red) and −100 mV (blue). (B) Current-voltage plots of normalized stationary photocurrents of BR-WT evoked by continuous green light. For each cell, the stationary current amplitude at 0 mV was used for normalization. The dashed line is simply drawn to guide the eye. (C, D, G) Green light-induced stationary and blue laser flash-induced transient currents of BR-WT recorded at 0 mV (C), −30 mV (D) and −100 mV (G), green light illumination is indicated by a grey bar above the current traces. According to the illumination scheme above panel (C), the signals shown in (C,D,G) are superpositions of 12 recordings, from which the first is drawn in red color. In each sweep of the protocol, two blue laser flashes (indicated as #1, #1′… #12, #12′ for the 12 traces) were applied: The first blue flash was given at time Δt = 25 ms after the start of illumination with green light, the second at Δt = 25 ms after illumination stop. From sweep to sweep, Δt increased by 25 ms up to 300 ms. (E) and (F) show the transient currents in response to blue laser flash #1 (trace1) and #12 (trace 12) during illumination with green light from panel (C) in higher magnification.

**Figure 3:**
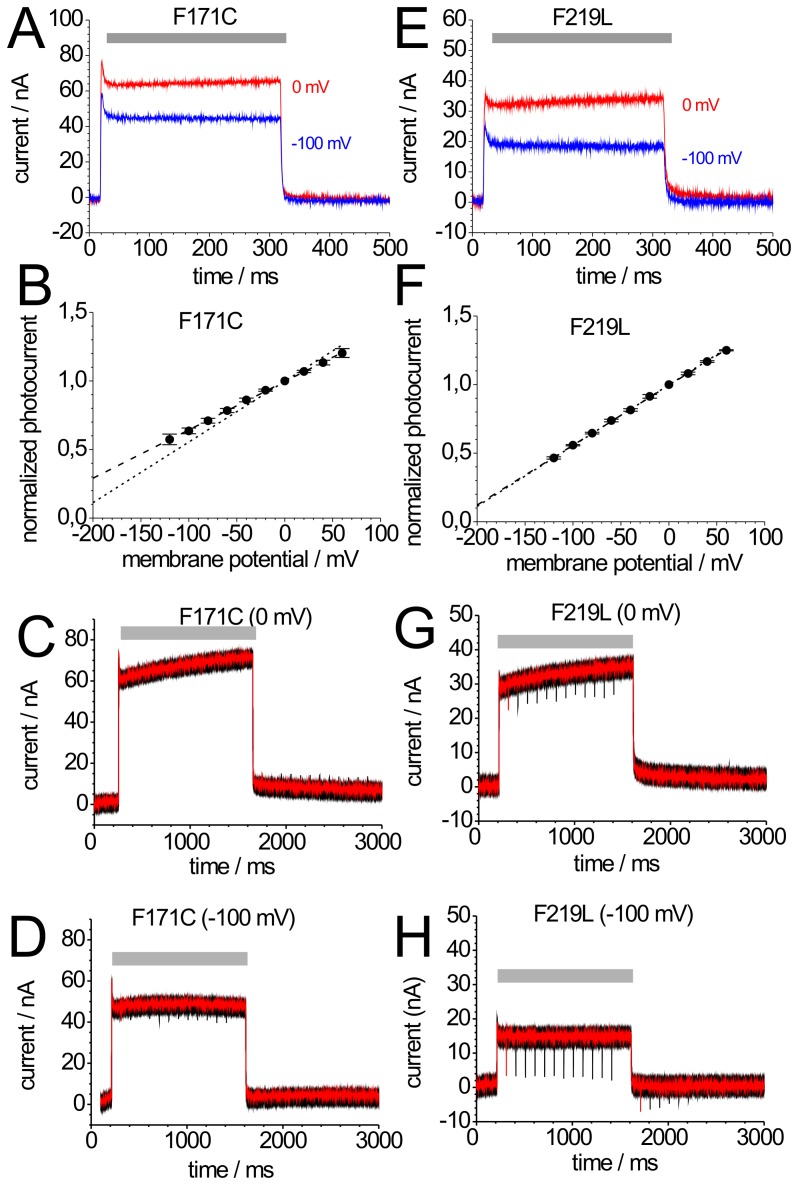
Photocurrents of mutants BR-F171C and BR-F219L. Photocurrents of BR-F171C (A) and BR-F219L (E) induced by illumination with green light (grey bar) at 0 mV (red) and −100 mV (blue). (B,F) Current-voltage plots of normalized stationary photocurrents of BR-F171C (B) and BR-F219L (F) evoked by continuous green light. For each cell, the stationary current amplitude at 0 mV was used for normalization. The dashed lines connecting the data points are drawn to guide the eye; for comparison, the corresponding WT curve from Fig. 2B is included as dotted line. (C,D,G,H) Green light-induced stationary and blue laser flash-induced transient currents of BR-F171C at 0 mV (C) and −100 mV (D), and BR-F219L at 0 mV (G) and −100 mV (H). Green light illumination is indicated by grey bars. The shown signals are superpositions of 12 recordings according to the illumination protocol from Fig. 2C. In each sweep, the first blue flash was given at Δt = 100 ms after start and the second at Δt = 100 ms after the end of illumination with green light. From sweep to sweep, Δt increased by 100 ms up to 1200 ms.

**Figure 4:**
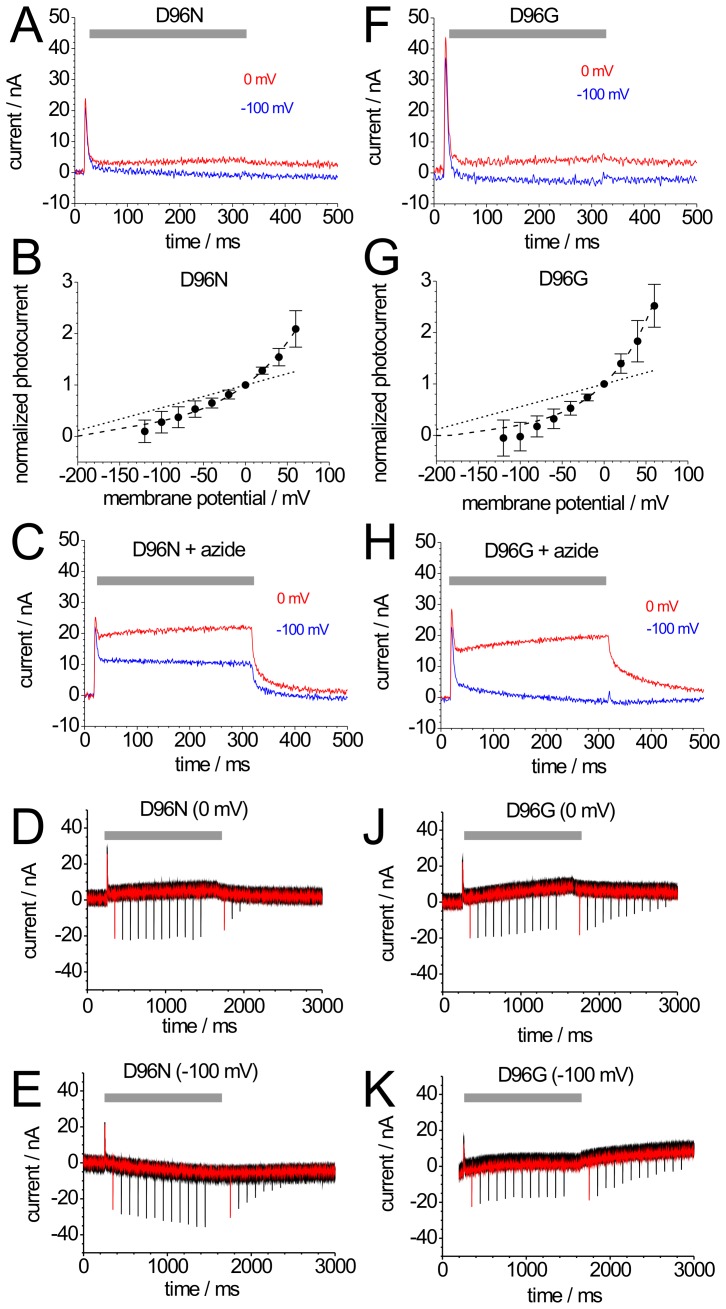
Photocurrents of mutants BR-D96N and BR-D96G. Photocurrents of BR-D96N (A) and BR-D96G (F) induced by illumination with green light (grey bar) at 0 mV (red) and −100 mV (blue). (B,G) Current-voltage plots of normalized stationary photocurrents of BR-D96N (B) and BR-D96G (G) evoked by continuous green light. For each cell, the stationary current amplitude at 0 mV was used for normalization. The dashed lines connecting the data points are drawn to guide the eye; for comparison, the corresponding WT curve from Fig. 2B is included as dotted line. (C,H) Photocurrents of BR-D96N (C, same cell as in panel A) and BR-D96G (H, same cell as in panel F) after addition of 50 mM azide. (D,E,J,K) Green light-induced stationary and blue laser flash-induced transient currents of BR-D96N at 0 mV (D) and −100 mV (E), and of BR-D96G at 0 mV (J) and −100 mV (K). Green light illumination is indicated by grey bars. The shown signals are superpositions of 12 recordings according to the illumination protocol from Fig. 2C. In each sweep, the first blue flash was given at Δt = 100 ms after start and the second at Δt = 100 ms after the end of illumination with green light. From sweep to sweep, Δt increased by 100 ms up to 1200 ms.

**Figure 5:**
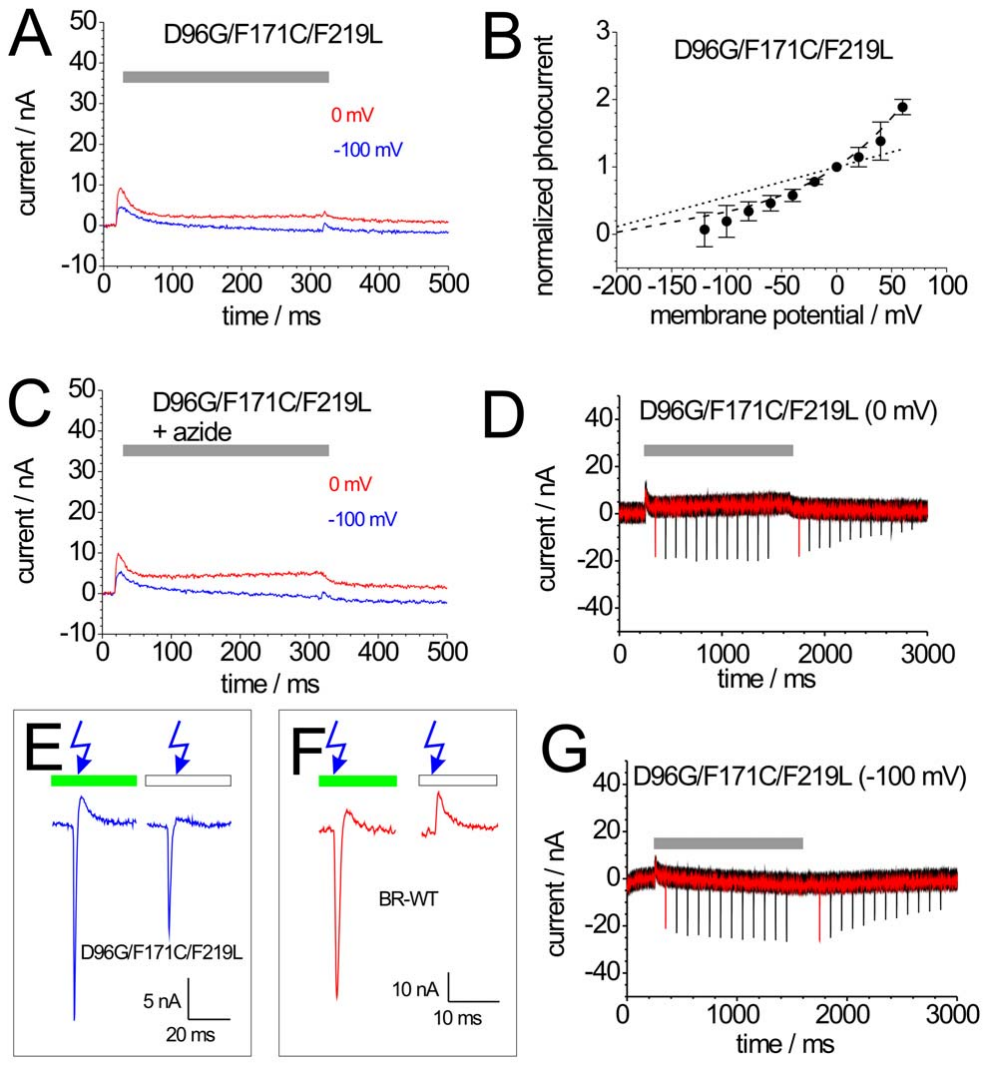
Photocurrents of mutant BR-D96G/F171C/F219L (BR-tri). (A) Photocurrents of BR-tri induced by illumination with green light (grey bar) at 0 mV (red) and −100 mV (blue). (B) Current-voltage plot of normalized stationary photocurrents evoked by continuous green light. For each cell, the stationary current amplitude at 0 mV was used for normalization. The dashed line connecting the data points are drawn to guide the eye; for comparison, the corresponding WT curve from Fig. 2B is included as dotted line. (C) Photocurrents of BR-tri (same cell as in panel A) after addition of 50 mM azide. (D,G) Green light-induced stationary and blue laser flash-induced transient currents of BR-tri at 0 mV (D) and −100 mV (G). Green light illumination is indicated by grey bars. The shown signals are superpositions of 12 recordings according to the illumination protocol from Fig. 2C. In each sweep, the first blue flash was given at Δt = 100 ms after start and the second at Δt = 100 ms after the end of illumination with green light. From sweep to sweep, Δt increased by 100 ms up to 1200 ms. (E,F) Transient photocurrents of BR-tri (E) and BR-WT (F) in response to blue laser flashes in higher magnification. Blue laser flashes were either applied during continuous illumination with green light (left signals in E,F) or without illumination in the dark (right signals in E,F).

Upon expression of wild-type and mutant BR constructs in *Xenopus* oocytes, transient and stationary photocurrents were measured using the two-electrode voltage-clamp technique. Figure 1 shows the location of the mutated amino acids within the BR structure and illustrates, that all mutated residues are located within the intracellular part (with respect to the retinal) of the proton transport pathway. As observed for BR-WT (Fig. 2A), all investigated mutants respond to green light (λ >495 nm) with positive transient and stationary photocurrents consistent with outwardly directed H^+^ translocation (Fig. 3A,E; Fig. 4A,F; and Fig. 5A) indicating correct right-side-out insertion in the cellular membrane.

### 3.1. Stationary Photocurrents of BR-WT

Photocurrents of BR-WT in response to green light exhibit three phases, which can be attributed to distinct processes of the photocycle (given in brackets). Upon switching-on illumination, a positive transient current is observed (*BR*→M, on-response), which is followed by a positive stationary current (turnover cycling; “*BR*→*BR*”, or more correctly “M→M”), and after light switch-off the photocurrent decays to zero (M→*BR* decay, off-response) (Fig. 2A). As shown previously [1], [24], the off-response consists of two exponential components reflecting two time constants during the M→*BR* decay. The fast time constant (τ_f_ ∼ 1–2 ms) is apparently voltage-independent and can not be resolved due to the limited time resolution of about 1–2 ms in TEVC experiments. The slow time constant τ_s_ is approximately linearly dependent on voltage. It represents the rate-limiting step of the BR pump cycle (∼33 ms at 0 mV and pH 7.5 [1]) and therefore determines the stationary current. Figure 2A depicts photocurrents upon stationary illumination at 0 mV and –100 mV. The peak amplitudes are less voltage-dependent than the stationary currents, which can be attributed to a lower voltage dependence of the initial photocycle events (retinal isomerisation followed by transfer of the SB proton to Asp-85 and H^+^ release from the extracellular H^+^ release complex). The current-voltage (I-V) curve for the stationary photocurrents of BR-WT is linear between −120 mV and +60 mV (Fig. 2B). Unfortunately, the occurrence of time-dependent baseline drifts at voltages exceeding +60 mV or −120 mV precluded the determination of current amplitudes with satisfactory accuracy. Assuming that inversion of the BR photocycle and photocurrent reversal cannot occur, current amplitudes should decrease to zero in a non-linear, but monotonous fashion at more negative potentials. Conversely, at depolarizing potentials, non-linearity due to saturation of photocurrent amplitudes is expected from an enzyme kinetic point of view [25], [26].

Notably, non-linear decrease of stationary photocurrents at negative potentials was readily observed for M stabilizing mutants (Fig. 4B,G and Fig. 5B). The voltage dependence from slopes of the stationary I-V curves of the various BR constructs can be compared by linear extrapolation of the normalized current amplitudes (Fig. 2B) to the ‘potential of zero current’ (V_I = 0_), which is about –220 mV for BR-WT. Of note, V_I = 0_ should not be confused with the term ‘reversal potential’ that is used to characterize passively conducting ion channels. The more negative V_I = 0_ is for a particular mutant, the weaker is the voltage dependence of proton pumping.

### 3.2. Pre-steady State Photocurrents of BR-WT

BR in the M state absorbs blue light, whereupon the retinal re-isomerizes and reprotonation of the SB occurs from the extracellular side from Asp-85, thereby short-circuiting the photocycle, which is known as blue light quenching. Therefore, additional illumination with blue laser flashes (λ = 396 nm, 10 ns pulse duration) results in transient quenching of the stationary photocurrent, showing up as inwardly directed transient currents (Fig. 2C,D,G). Figure 2E,F shows such blue laser flash-induced currents (#1 and #12 from Fig. 2C) on an expanded time scale. After the blue flash-induced inward current peak, a small positive transient current is observed, which is due to the fact that those BR molecules, which had just been shot back to the ground state by the blue laser flash, are re-excited by the green background light.

The blue laser flash-induced transient currents are not fully resolved in time due to the system time constant of TEVC experiments, which limits the time constants for the fast rise and decay of the negative transients to ∼1.4 ms (Fig. 2E,F). Since these time constants are invariant from flash to flash, the peak amplitude is proportional to the amount of charge translocated during the transient current. In turn, the charge is a relative measure for the amount of BR molecules, which momentarily populate the M intermediate, since during blue light quenching in each BR molecule a proton is translocated across a fraction of the transmembrane field by traveling the distance between Asp-85 and the SB. Thus, the negative peaks of the blue laser flash-induced currents during illumination with green light indicate the amount of BR molecules in the M state.

The blue laser flash-induced negative transient currents in Fig. 2C show the formation and decay of M for wild-type BR at 0 mV. The graph represents an overlay of 12 traces with different time intervals n·Δt (25 ms to 300 ms) between the start or the end of stationary illumination and the blue laser flash. Blue laser flashes applied during illumination resulted in large negative peak currents, which did not depend on Δt during stationary illumination (see also Fig. 2E: Δt = 25; Fig. 2F: Δt = 300 ms). For wild-type BR at 0 mV, the negative peak disappears already shortly after illumination with green light, because BR molecules rapidly decay to the ground state (Fig. 2C). After long intervals in the dark, no M intermediate is detectable and blue light flashes only result in positive transient photocurrents (see also Fig. 5F), which is due to residual blue light absorption of BR molecules in the ground state, as shown previously ([1], Fig. 3). Whereas the stationary currents decrease, the negative transient currents induced by blue laser flashes during illumination with green light increase at negative potentials (Fig. 2D,G) indicating increased accumulation of M. This is supported by the pronounced blue flash-induced negative transients after the end of stationary illumination with green light at −30 (Fig. 2G) and −100 mV (Fig. 2F), which reveal substantial accumulation and drastically slowed M decay, with typical time constants of several tens to hundreds of ms, as shown previously [1], [24].

### 3.3. N-accumulating Mutants (1): BR-F171C

Fig. 3A depicts photocurrent signals of mutant BR-F171C at 0 mV and −100 mV induced by green light. Stationary currents reach ∼100 nA at 0 mV, the same magnitude as BR-WT. Also the transient on- and the exponentially decaying off-response are clearly visible. However, the voltage sensitivity of the BR-F171C stationary photocurrent is weaker than that of BR-WT, with shallower slope of the I-V curve (Fig. 3B) and a more negative V_I = 0_ (−280 mV).

**Figure 6:**
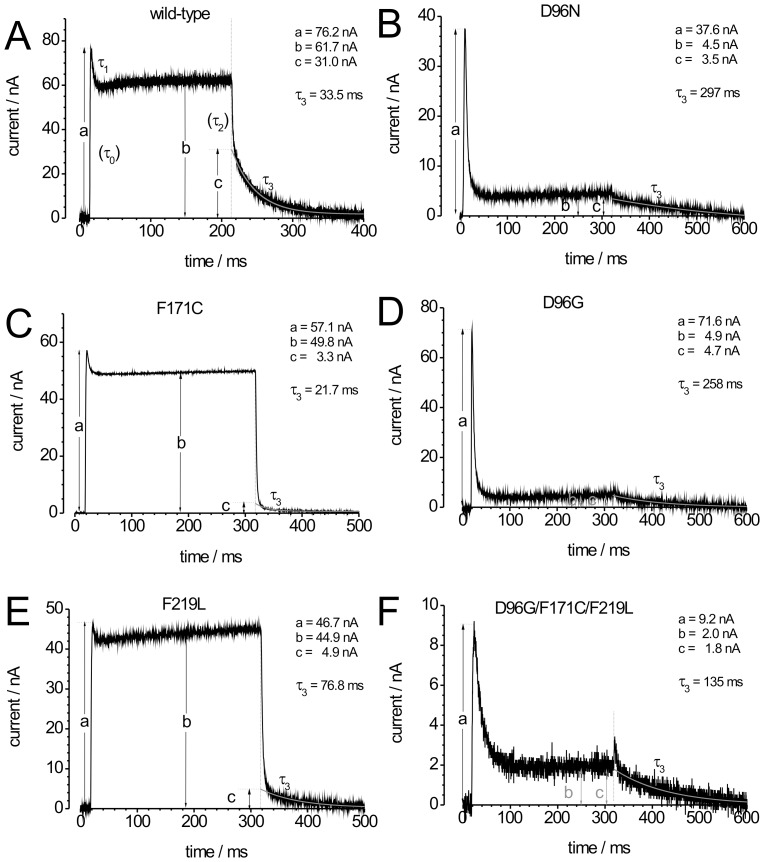
Comparison of characteristic properties of photocurrent signals. Photocurrents in response to continuous green light are characterized by five parameters: the amplitude of the transient current peak at the beginning of illumination (a), a stationary current amplitude (b), an initial amplitude of the slow phase of current decay after the end of illumination (c), a relaxation time τ_1_ for the decrease from the initial peak current to the stationary level and τ_3_ for the slow current decay after the end of illumination. The time constants for the initial current increase (τ_0_) at the beginning and for the initial current decrease after light switch-off (τ_2_) are not resolved due to the limited time resolution in TEVC experiments. In each panel, the parameters for the shown signals are included for comparison.

The transient current preceding the stationary current reflecting the reaction *BR*→M [1], is similar to BR-WT. The stationary current amplitude is determined by the reactions M_1_→M_2_→BR, as long as BR→M is much faster. Changes in the kinetics of these reactions would strongly influence the I_peak_/I_stationary_ ratio. However, the I_peak_/I_stationary_ value of BR-F171C is comparable to that of BR-WT indicating a similarly fast photocycle. The decay phase (off-response) of mutant BR-F171C exhibits two exponential components with time constants similar to BR-WT (see Fig. 6) indicating that the rate-limiting step of the transport cycle is not significantly altered. These time constants agree well with the time constants of 3 ms and 8 ms determined at pH 7 by time-resolved FT-IR spectroscopy for the de- and reprotonation of Asp-96, respectively [27], [28]. In notable contrast to BR-WT, the fast component has a larger amplitude implying that the major fraction of BR-F171C molecules, which decay to the ground state in the dark reaction does not generate large charge movement. This observation on the N-accumulating BR-F171C mutant is in line with the interpretation that the electrogenicity of the N→O→BR partial reaction sequence is smaller than that of the preceding M→N transition. Blue laser flashes applied to BR-F171C during green illumination did not result in negative transient currents at 0 mV (Fig. 3C), and at −100 mV negative transients were only small (Fig. 3D) indicating that the accumulation of M intermediates during illumination is drastically reduced. This property of BR-F171C (indicating that SB reprotonation proceeds faster to bring about M decay in favor of N) is remarkable considering that Phe-171 resides at the largest distance to the SB from all amino acids studied herein.

### 3.4 N-accumulating Mutants (2): BR-F219L

Figure 3E shows photocurrents of mutant BR-F219L at 0 mV and –100 mV upon illumination with green light. Stationary photocurrent amplitudes reach 30–50% of BR-WT currents, whereas I_peak_/I_stationary_ values are comparable (Fig. 6). The slope if the stationary I-V curve and V_I = 0_ (–228 mV), is almost identical to BR-WT (Fig. 3F). The slow time constant from the stationary photocurrent’s off-response is similar to BR-WT, but with smaller relative amplitude, even though it is slightly larger than that of BR-F171C (Fig. 6). Blue laser flashes induce smaller negative peak currents during green-light illumination at 0 mV (Fig. 3G) indicating reduced accumulation of M, the effect is weaker compared to BR-F171C. Blue flash-induced peak currents of mutant BR-F219L during green illumination increase strongly at −100 mV (Fig. 3H), and after green illumination the negative peaks decrease very slowly indicating that for BR-F219L negative potentials even more profoundly affect M decay than for BR-WT.

### 3.5. M-accumulating Mutants (1): BR-D96N

Photocurrents of mutant BR-D96N at 0 mV and –100 mV are at least 20 times smaller compared to BR-WT (Fig. 4A), whereas transient peaks (on-response) still reach ∼25% of the BR-WT amplitudes. The stationary photocurrents vanish almost completely at –100 mV, and the I_peak_/I_stationary_ ratio is strongly altered (Table 1), indicating a drastically slowed photocycle. This is confirmed by a similarly increased time constant of the photocurrent’s off-response (Fig. 6). The decay phase is less well defined due to the small amplitudes, and only single-exponential fits can be performed yielding time constants of ∼200 ms at 0 mV (Fig. 6) indicating that the rate-limiting step of the entire pumping process is dramatically slowed. Moreover, the I-V curve of BR-D96N (Fig. 4B) is clearly non-linear and steeper between 0 mV and +60 mV compared to BR-WT. The asymptotic decrease of the I-V curve to zero demonstrates that the vectoriality cannot be inverted simply by applying sufficiently large negative voltages.

**Table 1:** Properties of BR wild-type and mutants.

Construct (# of oocytes)	BR-WT (N = 5)	BR-F171C (N = 6)	BR-F219L (N = 4)	BR-D96N (N = 6)	BR-D96G (N = 5)	BR-tri (N = 3)
mean stationary current I_stat_ in nA	95.7±17.6	51.6±13.0	40.2±11.7	3.1±1.7	2.7±1.4	2.9±0.6
mean peak current I_peak_ in nA	125.0±22.1	59.7±16.1	44.0±12.4	31.2±6.5	52.3±37.7	9.7±2.9
I_peak_/I_stat_ (at 0 mV)	1.31±0.17	1.15±0.04	1.11±0.15	11.5±3.4	20.6±14.9	3.4±1.2
apparent potential of zerocurrent (V_I = 0_) in mV	−222	−280	−228	n.d.*	n.d.*	n.d.*
M decay τ_2_ (at 0 mV) in ms(from off response)	27.2±2.2	30.9±2.4	42.3±6.9	>300	>300	>300
# of molecules in oocytemembrane (see Equation 1)	1.6·10^10^	1.0·10^10^	1.1·10^10^	>5.8·10^9^	>5.0·10^9^	>5.4·10^9^
M decay τ_3_ (at 0 mV) in ms (from blue laser flash experiments)	<100	<100	<100	>300	>1200	>1500

* n.d. - not determined.

Blue laser flash experiments for mutant BR-D96N elicited larger negative transient currents already at 0 mV (Fig. 4D) compared to both, the stationary currents and the blue laser flash-induced transient currents of BR-WT. After the end of green light illumination, the M decay occurred with a much slower time constant at 0 mV (Fig. 4D) compared to BR-WT (∼300 ms vs. ∼100 ms), and was even slower (>1000 ms) at –100 mV (Fig. 4E), similar to previous observations [1], [24].

### 3.6. M-accumulating Mutants (2): BR-D96G

Stationary green light-induced photocurrents of mutant BR-D96G were comparable to BR-D96N (compare Fig. 4F to 4A), with a similar I-V curve that also decayed asymptotically to zero at negative potentials (Fig. 4G). Blue laser flash experiments again showed large accumulation of M during illumination with green light. The decrease of the blue flash-induced peak currents after illumination with green light at 0 mV was even slower than for BR-D96N (Fig. 4J; ∼1200 ms), but did not increase as profoundly at –100 mV (Fig. 4K).

### 3.7. Azide Effects on BR-D96N and BR-D96G

As expected from previous studies [29], azide (20 mM) greatly restores normal proton pump activity in mutants BR-D96N (Fig. 4C) and BR-D96G (Fig. 4H), which can be seen from the strong increase of stationary photocurrents and the decreased time constants of the photocurrents’ off-responses (Fig. 2G). Blue laser flashes also revealed a faster M decay in the presence of 20 mM azide (data not shown). Thus, also in the *Xenopus* oocyte system, the drastically reduced rate of proton pumping by mutants BR-D96N and BR-D96G can be recovered by the addition of azide, with the notable difference that the stationary photocurrent amplitudes of BR-D96G in the presence of azide at –100 mV are more profoundly decreased than for BR-D96N.

### 3.8 M-accumulating Mutants (3): BR-D96G/F171C/F219L

Photocurrents observed for the mutant BR-D96G/F171C/F219L (BR-tri) in response to continuous illumination with green light resemble those of mutants BR-D96G and BR-D96N. Following a comparatively large transient current, stationary currents are very small (Fig. 5A) and exhibit a slow M decay (from off-response). Similar to BR-D96N and BR-D96G, the I-V curve is steeper than for BR_WT_ in the range between 0 mV and +60 mV and asymptotically decays to zero at negative potentials (Fig. 5B). Some enhancement of the stationary photocurrent occurs upon addition of 20 mM azide (Fig. 5C), but by far not as strong as for the two Asp-96 mutants. Blue laser flash experiments revealed a high degree of M accumulation at 0 mV (Fig. 5D), similar to BR-D96G and BR-D96N, but only small increases of the negative peak currents were observed at –100 mV (Fig. 5G), indicating reduced voltage sensitivity of M accumulation in BR-tri.

The feature that distinguishes BR-tri from all single mutants studied in this work is that negative, inwardly directed transient currents in response to blue laser flashes are observed even after long times in the dark (Fig. 5E). This is in contrast to BR-WT, for which blue laser flashes without green background illumination exclusively induced positive transient currents (Fig. 5F), as expected for single-turnover forward transport caused by residual absorption of blue light.

## Discussion

Photocurrents of several structurally and functionally important BR mutants were investigated in *Xenopus* oocytes under membrane potential control. Mutants BR-D96G and BR-D96N had already been studied in black lipid membrane experiments or with purple membranes oriented in polyacrylamide gels [30], [31]), in which no transmembrane potential control is possible. All mutants generated positive photocurrents indicating outwardly directed proton pumping, but large differences in photocurrent amplitudes, voltage dependence and kinetics were observed.

### 4.1. Classification of the BR Mutants by Voltage Dependence of Photocurrents and M Lifetime

The first group of mutants is represented by BR-F171C and BR-F219L, which destabilize M in favor of the N intermediate. The photocurrents of these mutants are approximately of the same size as BR-WT currents, but the voltage sensitivity within the +60 mV to −120 mV range studied here is reduced for BR-F171C, as demonstrated by the mutant’s shallower I-V curve, and essentially unchanged for BR-F219L. An analogous behavior is found for the accumulation of the M intermediate by these mutants. For the mutant with the weakest voltage dependence, BR-F171C, no significant accumulation of M could be found, as indicated by the absence of negative blue laser flash-induced transient currents. For BR-F219L, still a smaller amount of M could be detected (compared to BR-WT). The lack of detectable M for BR-F171C should not imply that no M is formed during the photocycle. Rather, the M intermediates are too short-lived so that measurable accumulation of M is prohibited. Regarding the electrogenicity of the reaction steps during the M→BR branch, it is important to note that the time constants of BR-F171C from the decay of the stationary current are similar to those of BR-WT (see Fig. 6) indicating that the rate-limiting step of the transport cycle is not significantly altered. However, in contrast to BR-WT, the fast component is much larger than the slow one showing that a major fraction of BR-F171C molecules decaying to the ground state after the end of steady-state illumination does not generate large charge movement. Since BR-F171C in known to accumulate N, these observations suggest that the electrogenicity of the N→O→BR partial reaction sequence is small compared to that of the preceding M→N transition.

The differential amount of M found for mutants BR-F171C and BR-F219L under identical illumination conditions indirectly suggests a differential degree of N accumulation, which is notable since both mutants were used to determine the 3D structure of the N intermediate. The results from our electrophysiological experiments can be reconciled with findings from X-ray crystallographic studies. In a study using BR-F171C it was claimed that all protein molecules could be driven into the N state [4], [5], whereas in another study on BR-F219L [12], only 33% of the pump molecules were found to be present in the N state upon illumination, while the rest remained in the parental state [32]. The incomplete enrichment of N in BR-F219L was attributed to the large conformational changes suggesting that only one pump molecule per trimer in a purple membrane array could undergo the required large conformational change to avoid steric clashes. The BR-F219L molecules in the plasma membrane of oocytes (see Table 1∶10^10^ molecules per cell, i.e. 3300 molecules per µm^2^ for a spherical cell with 1.5 mm diameter) do not achieve such a dense packing comparable to the situation in native purple membranes. Thus, it seems unlikely that the lower population of the N state in mutant BR-F219L is a consequence of steric hindrance. Rather, the reduced accumulation of N should be regarded as a characteristic kinetic property of this mutant H^+^ pump.

The second group consists of mutants BR-D96N, BR-D96N and BR-tri (BR-D96G/F171C/F219L), which all are known to accumulate M strongly during illumination [8], [30], [31]. All these mutants consistently show largely reduced stationary photocurrents (at least by a factor of 20). This is accompanied by similarly increased decay time constants of the photocurrent’s off-response at an extracellular pH of 7 (to ∼250 ms, Table 1), which according to previous work represents the rate-limiting step of the photocycle [1]. BR-D96N, BR-D96G, and BR-tri show a large M accumulation during illumination as indicated by large negative transient currents upon blue laser flashes. This is paralleled by a drastically increased lifetime of M after illumination compared to BR-WT, reaching from ∼300 ms for BR-D96N over ∼800 ms for BR-D96G to >1000 ms for BR-tri (pH 7). These values are in good agreement with published data from spectroscopic measurements (BR-D96N, BR-D96G >500 ms; [30]). Regarding the nature of the M intermediate(s) probed by blue laser flashes, one can refer to a structural study by Luecke et al. [8], which attributed conformational changes in BR-D96G to an early M intermediate such as M_1_, while the structural changes in BR-D96N were proposed to represent formation of a later intermediate M_N_, which is similar to M_2_. The major difference between M_1_ and M_2_ is the direction of SB accessibility. Similar to the effect of mutations on the M_1_/M_2_ distribution, the orientation of the SB can be changed by the electrical field, as shown for BR-WT [24]. The exclusively negative amplitudes of blue laser flash-induced transient currents show that SB reprotonation upon blur light only occurs from the extracellular side (Asp-85), independent from the previous SB orientation (either promoted by mutation or by the electrical field). Most likely, after the absorption of a blue photon in the M state, the retinal isomerizes back to *all-trans* with SB accessibility to the EC part of the proton translocation pathway.

It is important to note, that the observed small photocurrents are not due to a greatly reduced expression level. The number of BR molecules in the plasma membrane of oocytes can be estimated as follows: The slow phase of the stationary current’s off-response (∼27 ms at 0 mV for BR-WT) constitutes the rate-limiting step for the whole photocycle, and stationary turnover cannot proceed faster than the corresponding rate constant (37 s^-1^). Therefore, a lower limit for the number *N* of BR molecules which gives rise to the observed stationary current can be calculated by the following equation:
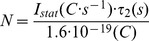
(1)where *I_stat_* is the stationary current amplitude and τ_2_ the rate-limiting time constant. The resulting *N* values are given in Table 1 for all studied constructs and show that the expression levels only vary by a factor of ∼2–3. This variation cannot account for the drastically reduced stationary currents observed for M-stabilizing mutants (by more than a factor 30). M-accumulating mutants are simply weak proton pumps, and this should also impair the ability to generate an electrochemical H^+^ gradient within a cellular context. At negative voltages, the I-V curves of the M-accumulating mutants are non-linear and approach the zero current line asymptotically. This in accord with the notion that the vectoriality of the light-driven H^+^ pump BR cannot be inverted simply by applying sufficiently negative voltages. The limited voltage range accessible by TEVC so far precluded the observation of such a behavior of BR-WT.

### 4.2. The Role of the M Intermediates for the Voltage Sensitivity of Proton Pumping

The understanding of the voltage sensitivity of proton pumping in BR is an essential aspect of the molecular mechanism. Our results show that the life-time of the M intermediates determines the current-voltage behavior of BR and, therefore, serves in the regulation of proton pumping. Mutants BR-D96N, BR-D96G and BR-tri achieve M accumulation by a drastically increased M lifetime. Mutants BR-F171C and BR-F219L accumulate N by a faster M_2_ decay and not by a slowed N decay, since the time constants for the photocurrent’s off-response are not significantly changed compared to BR-WT. It has been shown that the voltage dependence of stationary proton pumping by BR is regulated by two effects, which are related to the life-time of the M intermediate. The main electrogenic event occurs during the M→N→O→*BR* branch of the photocycle, which is reflected by the pronounced voltage dependence of the slow decay constant (τ_2_) of the stationary current’s off-response. The voltage dependence of τ_2_ is stronger than that of the stationary I-V curve [1], and at strongly negative membrane potentials an additional, much slower M decay time constant becomes apparent. This has led us to propose a branching – most likely from M_1_– into a futile reaction pathway involving a still M-like intermediate, which also allows for reprotonation of M from the extracellular side [1], [24]. The extent to which this branching occurs, depends on the strongly voltage-dependent lifetime of M and explicitly implies reversibility of the M_1_↔M_2_ reaction [1]. At large negative potentials the M lifetime is drastically increased allowing more BR molecules to enter and accumulate in the futile reaction branch.

The strong effect of the lifetime of M on the voltage dependence of proton pumping is corroborated by the differential voltage sensitivity of the mutants studied here. The slopes of the I-V curves of mutants with undetectable (BR-F171C) or reduced (BR-F219L) M accumulation are shallower, and those of mutants with strong M accumulation (BR-D96G, BR-D96N and BR-tri) are steeper than in BR-WT. Accordingly, the increase of M lifetime probed by blue laser flashes follows the same order (BR-F171C ≤ BR-F219L<BR-WT<BR-tri<BR-D96N<BR-D96G). Thus, the profound correlation between voltage dependence of proton pumping and the lifetime of the M intermediate, as well as the augmentation of stationary pump currents of M accumulating mutants by azide is a characteristic feature of BR. These data are consistent with observations on sensory rhodopsins (SR) when expressed in *Xenopus* oocytes in the absence of their cognate transducer proteins. The SR proteins also exhibit long-lived M analogues during the photocycle together with a profound voltage-dependence and steep I-V curves and show substantial augmentation of stationary proton pumping by azide [33]. Of note, these findings do not contradict results from surface-enhanced infrared difference absorption spectroscopy (SEIDAS) studies on SRII from Halobacterium salinarum (or Natronomonas pharaonis) showing a pronounced inhibition of proton transfer from the retinal SB to the primary proton acceptor Asp-75 (or Asp-73, respectively) [34], which according to later work [35] is strongly dependent on pH, in accordance with a profound effect of the applied electric field on the protonation state of Asp-75. In our photocurrent measurements, the initial proton transfer step corresponds to the positive peak current preceding the stationary photocurrent, which also exhibits a marked, albeit weaker, voltage dependence, which was shown to be similarly dependent on the extracellular pH [1].

### 4.3. Proton Pumping in the Absence of Substantial Conformational Changes – the BR-Tri Mutant (BR-D96G/F171C/F219L)

The most severe changes in photocurrent properties compared to BR-WT are found for BR-tri. The current-voltage behavior of BR-tri is largely governed by the characteristic properties of BR-D96G, which removes the primary proton donor for reprotonation of the SB. Stationary photocurrents are very small (only ∼3% of BR_WT_ currents at 0 mV), which severely impairs the ability to pump protons. Again, the small photocurrents are consistent with increased time constants for the rate-limiting step (off-response of the stationary current, >300 ms) and cannot be due to a reduced expression level (see Table 1).

**Figure 7:**
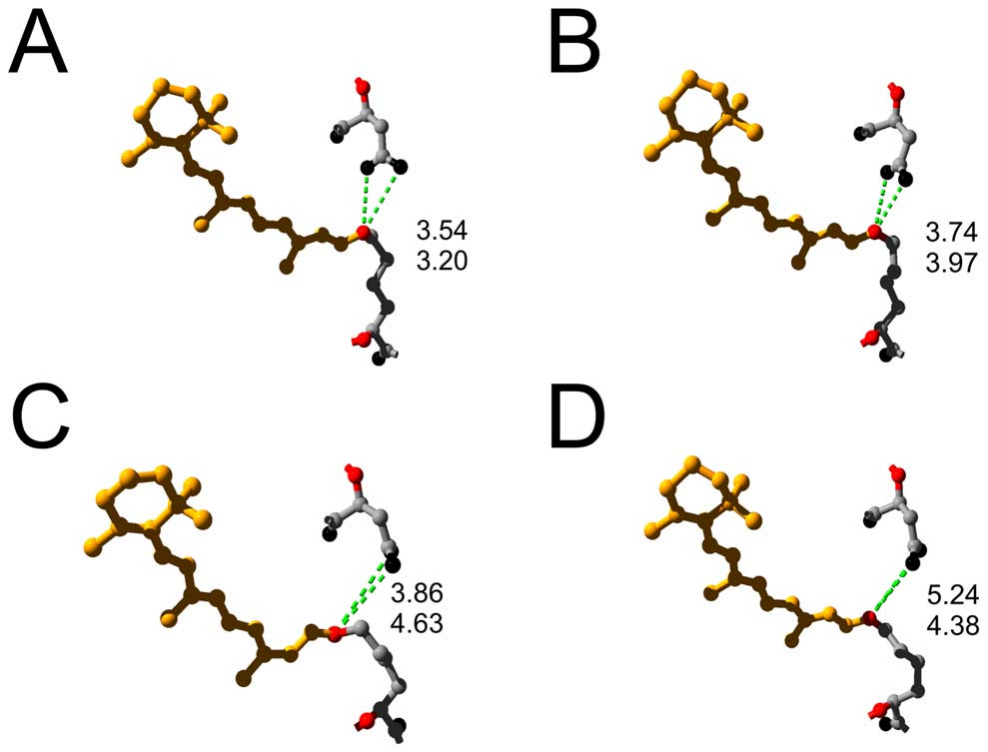
Structural details during the BR photocycle. Three-dimensional ball and stick representations of the retinal chromophore (yellow), coupled via a Schiff base to Lys-216 (nitrogen atom in red) are shown and distances from the Schiff base nitrogen to the carboxyl oxygens (black) of Asp-85 are indicated by green dashed lines according to the following structural coordinates: (A) BR ground state structure (PDB structure entry 1C3W) [9], (B) BR ground state structure (PDB structure entry 1FBB) [41], (C) structure of the M intermediate (PDB structure entry 1C3W) [9], (D) Ground state structure of the triple mutant BR-D96G/F171C/F219L (PDB structure entry 1FBK) [11].

Some features of the triple mutant, however, differ markedly from all other mutants. Firstly, the transient current (on-response) has a different shape compared to BR-WT. Rising and falling phase are slower, which can be interpreted as a slower initial phase of the photocycle (usually the formation of M). This is in line with the slower M formation, as determined by spectroscopy (1 ms, vs. 50 µs for BR-WT) in a study of Tittor and colleagues [14]. How can this behavior be explained? In Figure 7, the distances between the SB and the carboxyl oxygens of Asp-85 are depicted according to structural information for BR-WT and BR-tri. The structures provided by Sass et al. for BR-WT (ground state and M) showed that the distance increases during the *BR*→M transition (Fig. 7A,C). The BR-tri structure by Subramaniam et al. [11] revealed that (1) the distance between Asp-85 and the SB is increased in the unilluminated ground state already, and (2) that the CP channel is opened (Fig. 7B,D). The increased distance between Asp-85 and the SB may consequently slow down SB deprotonation towards Asp-85 and the formation of M in BR-tri. Furthermore, the primary proton donor for SB reprotonation is removed due to the D96G mutation, which most likely also entails a slowed-down SB reprotonation during the photocycle of BR-tri. Both properties, in effect, would make BR-tri a slow and inefficient proton pump.

Unfortunately, the crystal structure does not allow conclusions about the protonation state of the SB in BR-tri. In this respect, the second exclusive property, which distinguishes BR-tri from the other mutants, is remarkable. Blue laser flash illumination in the dark (i.e. irrespective of pre-excitation) leads to negative peak currents (see Fig. 5E). If these negative currents of BR-tri were due to the same process as in BR-WT (i.e. blue light quenching of M and reprotonation of a deprotonated SB from Asp-85) this would indicate that SB deprotonation occurs spontaneously in BR-tri and that a significant part of the BR-tri molecules would assume a state with the deprotonated SB even in the dark. However, vibrational spectroscopy has demonstrated that the SB in BR-tri is protonated in the dark state [14]. Therefore, it is conceivable that the negative transient current is due to a fast and transient proton release towards the “wrong” CP-oriented side, as a consequence of the increased distance to Asp-85 and the already opened CP channel. Similar negative transient current components upon blue laser flash illumination have also been reported for the BR homolog proteorhodopsin (PR) from γ-proteobacteria, although in the latter case, this is not due to an increased distance between the retinal SB and the primary proton acceptor group (Asp-97 in PR), but due to the fact that the pK_a_ of Asp-97 (PR) is around 7.2, so that about 50% of the prooton acceptor groups are protonated at neutral pH [36]. This futile side reaction may contribute to the low proton pumping efficiency, as does the substantially slowed SB reprotonation through the (already open) CP pathway, as judged from the time constants of the photocurrent’s off-response (indicating slowed M decay). However, whereas photocurrents of BR-D96N/G single mutants are substantially accelerated by azide, thereby restoring rather normal H^+^ pump function, BR-tri photocurrents are hardly augmented by azide, which also suggests that the disturbance of structure and dynamics of the H^+^ pump caused by the triple mutation is more severe than just the defect of slowed SB reprotonation through the CP channel.

It is notable that the triple mutant was found to show structural features of the M intermediate already in the (unexcited) ground state and that only minor conformational changes occur upon illumination [14]. This has raised a discussion about the necessity of structural changes for efficient proton translocation. In the same study, it was claimed that the BR-tri mutant reaches 66% of the WT protein’s overall transport activity [14] even in the absence of detectable structural changes. However, the turnover number of ∼2 s^−1^ for BR-tri (at pH 6) determined in the same publication [14] compared to the generally accepted turnover number of BR-WT (50–100 s^−1^ [37], and ∼37 s^−1^ determined in this study), matches well with the data provided in our study, which showed about 30-fold larger photocurrents of BR-WT compared to BR-tri. Thus, our results indicate that the proton pumping process of BR-tri under otherwise identical illumination conditions proceeds by a factor of at least 30 slower than in BR-WT, thus making BR-tri a profoundly weaker proton pump than the wild-type protein. In addition, these observations imply that the disruptive effect of the M-stabilizing D96G mutation, which *per se* results in a slow and inefficient proton pump, can not be compensated for by the N-stabilizing effect of the F171C (plus F219L) mutations.

### 4.4. Conformational Changes as Basis for Vectorial Transport of BR-WT

The properties of the BR mutants studied here suggest that potent vectorial ion transport by BR is intimately related to distinct, albeit comparatively subtle structural changes that need to occur in a temporally well coordinated manner. This mechanism determines correct sidedness of SB de- and reprotonation, and ensures vectoriality of H^+^ transport as well as efficient energy transduction. The available structures show that formation of M increases the distance between Asp-85 and the SB (compare Fig. 7A and 7C), which is comparable to the distance obtained for BR-tri in the ground state (Fig. 7B and 7D). In the early transport cycle intermediates, H^+^ transfer from the SB to Asp-85 is allowed, but the structural changes during M formation that increase the distance to Asp-85, prevent the proton from returning to the SB. By the same argument, the large distance between the SB and Aps-85 in the ground state of BR-tri accounts for the slowed on-response of the BR-tri photocurrents. Subsequently, the SB is reprotonated from Asp-96, and the large conformational changes of helices F, G and also helix B further facilitate reprotonation of Asp-96 through the CP channel. The conformational changes, the opening of the CP channel after deprotonation of the SB, and the change in its accessibility from EC to CP provide the appropriate framework for the capability of BR to efficiently pump protons.

## Materials and Methods

### 5.1. Ethics Statement

Surgical removal of ovary tissue from adult *Xenopus laevis* females followed registered protocols approved by the responsible state authority (Landesamt für Gesundheit und Soziales [LAGeSo] Berlin, Reg. No. O 0308/06) and the local ethics committee (Tierversuchskommission, Fachgruppe IC beim LAGeSo Berlin), in accordance with the German Animal Protection Act (Tierschutzgesetz). Animals were anesthetized by immersion in water containing 0.2% w/v tricaine (MS-222, Sigma, Deisenhofen, Germany) for 5 min, and subsequently placed on ice during surgical treatment. All efforts were made to minimize animal suffering.

### 5.2. cDNA Constructs

The *bacterio-opsin* (*bop*) cDNA was amplified from a pGEM-*bop* construct (kind gift of Dr. Phil G. Wood) using adapter primers adding *Nru*I and *Xba*I restriction sites 5′ and 3′ of the coding sequence, and inserting a Kozak sequence [38] immediately 5′ of the start codon. The fragment was cut with *Nru*I and *Xba*I and subcloned into vector pTLN [39]. Mutations were introduced using the QuickChange™ mutagenesis kit (Stratagene, La Jolla, CA) and verified by sequencing (Eurofins MWG Operon, Ebersberg, Germany).

### 5.3. Expression in *Xenopus laevis* Oocytes

After linearizing the plasmid with *Mlu*I capped cRNA prepared with SP6 RNA polymerase using the mMessage mMachine kit (Ambion, Austin, TX). Usually, 25 ng of cRNA were injected per cell. Oocytes were obtained by partial ovariectomy from anesthetized frogs and isolated by 3 hours treatment with collagenase 1A (Sigma, Deisenhofen, Germany) as described [40]. Oocytes were kept at 17°C in modified Barth’s solution (90 mM NaCl, 1 mM KCl, 0.41 mM CaCl_2_, 0.33 mM Ca(NO_3_)_2_, 0.82 mM MgSO_4_, 10 mM HEPES, 100 U penicillin–100 µg streptomycin/ml, pH 7.6) supplemented with 1 µM retinal.

### 5.4. Electrophysiology

BR photocurrents were recorded in the TEVC configuration on *Xenopus* oocytes with a GeneClamp500 amplifier (Molecular Devices, Union City, CA) or a Turbotec 10 CX amplifier (npi electronics, Tamm, Germany). Data acquisition, shutter triggering and control of transmembrane potential were performed with pClamp 7 or 9 software via a Digidata 1200B or Digidata 1440 interface (Molecular Devices, Union City, CA). Current traces were usually recorded at 5 kHz after filtering to 1 kHz using the amplifiers’ filtering circuits. Data traces obtained were usually averages of 3 runs. Since the observed photocurrents are quite small (<100 nA), changes in the leak conductances of the oocytes (1–10 µS) in the range of 1% lead to changes in offset currents of 1–10 nA over the potential range studied here. Therefore, all currents were calculated as the difference between currents evoked by a specific voltage-pulse first with, then without illumination. Only experiments without recognizable drifts in the background conductance were taken for further analysis. All experiments were carried out using a Na-100 bath solution (containing in mM: 100 NaCl, 15 TEA·Cl, 2 CaCl_2_, 10 TRIS, pH 7.5) at room temperature (21°C) 3–5 days after injection. The pipette solutions contained 3 mM KCl. Typical pipette resistances were between 0.5 and 2 MΩ.

### 5.5. Optical Equipment

To induce photocurrents, cells were illuminated with light from a mercury arc lamp (Carl Zeiss, Göttingen, Germany, equipped with an Osram HBO 100 W bulb), filtered through a heat protection and a short-wavelength cutoff filter (Schott GG495 = “green light”), and coupled into an optical light-guide. Illumination was controlled by a fast shutter (Uniblitz LS6ZM2, Vincent Associates, Rochester, NY), which provided a response time of <1 ms. For single flash experiments, light from a XeCl excimer laser-pumped dye laser (10 ns pulse duration, equipped with dye PBBO, 396 nm = “blue light”) was used. Due to the lower time resolution in oocyte experiments, the peak amplitudes of blue laser flash-induced currents are generally smaller than obtained in HEK293 cells [1].

## References

[1] Geibel S, Friedrich T, Ormos P, Wood PG, Nagel G, et al (2001) The voltage-dependent proton pumping in bacteriorhodopsin is characterized by optoelectric behavior. Biophys J 81: 2059–2068. 11566778 10.1016/S0006-3495(01)75855-9 PMC1301679

[2] Henderson R, Baldwin JM, Ceska TA, Zemlin F, Beckmann E, et al (1990) Model for the structure of bacteriorhodopsin based on high-resolution electron cryo-microscopy. J Mol Biol 213: 899–929. 2359127 10.1016/S0022-2836(05)80271-2

[3] Henderson R, Unwin PN (1975) Three-dimensional model of purple membrane obtained by electron microscopy. Nature 257: 28–32. 1161000 10.1038/257028a0

[4] Kamikubo H, Kataoka M, Varo G, Oka T, Tokunaga F, et al (1996) Structure of the N intermediate of bacteriorhodopsin revealed by x-ray diffraction. Proc Natl Acad Sci U S A 93: 1386–1390. 8643641 10.1073/pnas.93.4.1386 PMC39947

[5] Kataoka M, Kamikubo H (2000) Structures of photointermediates and their implications for the proton pump mechanism. Biochim Biophys Acta 1460: 166–176. 10984598 10.1016/s0005-2728(00)00137-7

[6] Landau EM, Rosenbusch JP (1996) Lipidic cubic phases: a novel concept for the crystallization of membrane proteins. Proc Natl Acad Sci U S A 93: 14532–14535. 8962086 10.1073/pnas.93.25.14532 PMC26167

[7] Luecke H, Schobert B, Richter HT, Cartailler JP, Lanyi JK (1999) Structure of bacteriorhodopsin at 1.55 A resolution. J Mol Biol 291: 899–911. 10452895 10.1006/jmbi.1999.3027

[8] Luecke H, Schobert B, Richter HT, Cartailler JP, Lanyi JK (1999) Structural changes in bacteriorhodopsin during ion transport at 2 angstrom resolution. Science 286: 255–261. 10514362 10.1126/science.286.5438.255

[9] Sass HJ, Buldt G, Gessenich R, Hehn D, Neff D, et al (2000) Structural alterations for proton translocation in the M state of wild-type bacteriorhodopsin. Nature 406: 649–653. 10949308 10.1038/35020607

[10] Subramaniam S, Lindahl M, Bullough P, Faruqi AR, Tittor J, et al (1999) Protein conformational changes in the bacteriorhodopsin photocycle. J Mol Biol 287: 145–161. 10074413 10.1006/jmbi.1999.2589

[11] Subramaniam S, Henderson R (2000) Molecular mechanism of vectorial proton translocation by bacteriorhodopsin. Nature 406: 653–657. 10949309 10.1038/35020614

[12] Vonck J (2000) Structure of the bacteriorhodopsin mutant F219L N intermediate revealed by electron crystallography. Embo J 19: 2152–2160. 10811606 10.1093/emboj/19.10.2152 PMC384371

[13] Unwin PN, Henderson R (1975) Molecular structure determination by electron microscopy of unstained crystalline specimens. J Mol Biol 94: 425–440. 1236957 10.1016/0022-2836(75)90212-0

[14] Tittor J, Paula S, Subramaniam S, Heberle J, Henderson R, et al (2002) Proton translocation by bacteriorhodopsin in the absence of substantial conformational changes. J Mol Biol 319: 555–565. 12051928 10.1016/S0022-2836(02)00307-8

[15] Hampp N (2000) Bacteriorhodopsin as a Photochromic Retinal Protein for Optical Memories. Chem Rev 100: 1755–1776. 11777419 10.1021/cr980072x

[16] Yao B, Ren Z, Menke N, Wang Y, Zheng Y, et al (2005) Polarization holographic high-density optical data storage in bacteriorhodopsin film. Appl Opt 44: 7344–7348. 16353805 10.1364/ao.44.007344

[17] Yao B, Lei M, Ren L, Menke N, Wang Y, et al (2005) Polarization multiplexed write-once-read-many optical data storage in bacteriorhodopsin films. Opt Lett 30: 3060–3062. 16315721 10.1364/ol.30.003060

[18] Fischer T, Neebe M, Juchem T, Hampp NA (2003) Biomolecular optical data storage and data encryption. IEEE Trans Nanobioscience 2: 1–5. 15382416 10.1109/tnb.2003.810163

[19] Der A, Valkai S, Fabian L, Ormos P, Ramsden JJ, et al (2007) Integrated optical switching based on the protein bacteriorhodopsin. Photochem Photobiol 83: 393–396. 17132043 10.1562/2006-06-21-RA-944

[20] Fabian L, Heiner Z, Mero M, Kiss M, Wolff EK, et al (2011) Protein-based ultrafast photonic switching. Opt Express 19: 18861–18870. 21996828 10.1364/OE.19.018861

[21] Mathesz A, Fabian L, Valkai S, Alexandre D, Marques PV, et al (2013) High-speed integrated optical logic based on the protein bacteriorhodopsin. Biosens Bioelectron 46C: 48–52. 10.1016/j.bios.2013.02.022 23500476

[22] Zhang J, Yamazaki Y, Hikake M, Murakami M, Ihara K, et al (2012) Crystal structure of the O intermediate of the Leu93–>Ala mutant of bacteriorhodopsin. Proteins 80: 2384–2396. 22641602 10.1002/prot.24124

[23] Nagel G, Mockel B, Buldt G, Bamberg E (1995) Functional expression of bacteriorhodopsin in oocytes allows direct measurement of voltage dependence of light induced H+ pumping. FEBS Lett 377: 263–266. 8543064 10.1016/0014-5793(95)01356-3

[24] Nagel G, Kelety B, Mockel B, Buldt G, Bamberg E (1998) Voltage dependence of proton pumping by bacteriorhodopsin is regulated by the voltage-sensitive ratio of M1 to M2. Biophys J 74: 403–412. 9449340 10.1016/S0006-3495(98)77797-5 PMC1299392

[25] Hagedorn R, Gradmann D, Hegemann P (2008) Dynamics of voltage profile in enzymatic ion transporters, demonstrated in electrokinetics of proton pumping rhodopsin. Biophys J 95: 5005–5013. 18621842 10.1529/biophysj.107.125260 PMC2586558

[26] Tsunoda SP, Ewers D, Gazzarrini S, Moroni A, Gradmann D, et al (2006) H+ -pumping rhodopsin from the marine alga Acetabularia. Biophys J 91: 1471–1479. 16731558 10.1529/biophysj.106.086421 PMC1518632

[27] Kotting C, Gerwert K (2005) Proteins in action monitored by time-resolved FTIR spectroscopy. Chemphyschem 6: 881–888. 15884070 10.1002/cphc.200400504

[28] Radu I, Schleeger M, Bolwien C, Heberle J (2009) Time-resolved methods in biophysics. 10. Time-resolved FT-IR difference spectroscopy and the application to membrane proteins. Photochem Photobiol Sci 8: 1517–1528. 19862409 10.1039/b9pp00050j

[29] Tittor J, Soell C, Oesterhelt D, Butt HJ, Bamberg E (1989) A defective proton pump, point-mutated bacteriorhodopsin Asp96-Asn is fully reactivated by azide. Embo J 8: 3477–3482. 2555165 10.1002/j.1460-2075.1989.tb08512.x PMC401504

[30] Tittor J, Soell C, Oesterhelt D, Butt HJ, Bamberg E (1989) A defective proton pump, point-mutated bacteriorhodopsin Asp96–-Asn is fully reactivated by azide. Embo J 8: 3477–3482. 2555165 10.1002/j.1460-2075.1989.tb08512.x PMC401504

[31] Butt HJ, Fendler K, Bamberg E, Tittor J, Oesterhelt D (1989) Aspartic acids 96 and 85 play a central role in the function of bacteriorhodopsin as a proton pump. Embo J 8: 1657–1663. 2548851 10.1002/j.1460-2075.1989.tb03556.x PMC401006

[32] Vonck J (1996) A three-dimensional difference map of the N intermediate in the bacteriorhodopsin photocycle: part of the F helix tilts in the M to N transition. Biochemistry 35: 5870–5878. 8639548 10.1021/bi952663c

[33] Schmies G, Engelhard M, Wood PG, Nagel G, Bamberg E (2001) Electrophysiological characterization of specific interactions between bacterial sensory rhodopsins and their transducers. Proc Natl Acad Sci U S A 98: 1555–1559. 11171989 10.1073/pnas.031562298 PMC29295

[34] Jiang X, Zaitseva E, Schmidt M, Siebert F, Engelhard M, et al (2008) Resolving voltage-dependent structural changes of a membrane photoreceptor by surface-enhanced IR difference spectroscopy. Proc Natl Acad Sci U S A 105: 12113–12117. 18719097 10.1073/pnas.0802289105 PMC2527874

[35] Jiang X, Engelhard M, Ataka K, Heberle J (2010) Molecular impact of the membrane potential on the regulatory mechanism of proton transfer in sensory rhodopsin II. J Am Chem Soc 132: 10808–10815. 20681714 10.1021/ja102295g

[36] Lörinczi E, Verhoefen MK, Wachtveitl J, Woerner AC, Glaubitz C, et al (2009) Voltage- and pH-dependent changes in vectoriality of photocurrents mediated by wild-type and mutant proteorhodopsins upon expression in Xenopus oocytes. J Mol Biol 393: 320–341. 19631661 10.1016/j.jmb.2009.07.055

[37] Müller K-H, Butt HJ, Bamberg E, Fendler K, Hess B, et al (1991) The reaction cycle of bacteriorhodopsin: an analysis using visible absorption, photocurrent and infrared techniques. Eur Biophys J 19: 241–251.

[38] Kozak M (1984) Compilation and analysis of sequences upstream from the translational start site in eukaryotic mRNAs. Nucleic Acids Res 12: 857–872. 6694911 10.1093/nar/12.2.857 PMC318541

[39] Lorenz C, Pusch M, Jentsch TJ (1996) Heteromultimeric CLC chloride channels with novel properties. Proc Natl Acad Sci U S A 93: 13362–13366. 8917596 10.1073/pnas.93.23.13362 PMC24098

[40] Grygorczyk R, Hanke-Baier P, Schwarz W, Passow H (1989) Measurement of erythroid band 3 protein-mediated anion transport in mRNA-injected oocytes of Xenopus laevis. Methods Enzymol 173: 453–466. 2674617 10.1016/s0076-6879(89)73032-9

[41] Subramaniam S, Henderson R (1999) Electron crystallography of bacteriorhodopsin with millisecond time resolution. J Struct Biol 128: 19–25. 10600554 10.1006/jsbi.1999.4178

